# Update on the genetics of paragangliomas

**DOI:** 10.1530/ERC-22-0373

**Published:** 2023-03-08

**Authors:** Anne-Paule Gimenez-Roqueplo, Mercedes Robledo, Patricia L M Dahia

**Affiliations:** 1 Université Paris Cité, PARCC, INSERM, Paris, France; 2 Département de Médecine Génomique des Tumeurs et des Cancers, Assistance Publique-Hôpitaux de Paris, Hôpital Européen Georges Pompidou, Paris, France; 3 Hereditary Endocrine Cancer Group, Human Cancer Genetics Programme, Spanish National Cancer Research Centre (CNIO), Madrid, Spain; 4 Biomedical Research Networking Centre on Rare Diseases (CIBERER), Institute of Health Carlos III, Madrid, Spain; 5 Division of Hematology and Medical Oncology, Department Medicine, University of Texas Health Science Center at San Antonio (UTHSCSA), San Antonio, Texas, USA; 6 Mays Cancer Center at UTHSCSA, San Antonio, Texas, USA

**Keywords:** pheochromocytoma, paraganglioma, susceptibility genes, germline mutations, somatic mutations

## Abstract

Paragangliomas (PGL) of the adrenal (also known as pheochromocytomas) or extra-adrenal neural crest-derived cells are highly heritable tumors, usually driven by single pathogenic variants that occur mutually exclusively in genes involved in multiple cellular processes, including the response to hypoxia, MAPK/ERK signaling, and WNT signaling. The discovery of driver mutations has led to active clinical surveillance with outcome implications in familial PGL. The spectrum of mutations continues to grow and reveal unique mechanisms of tumorigenesis that inform tumor biology and provide the rationale for targeted therapy. Here we review recent progress in the genetics and molecular pathogenesis of PGLs and discuss new prospects for advancing research with new disease models and ongoing clinical trials presented at the recent International Symposium of Pheochromocytomas and Paragangliomas (ISP2022) held in October 2022 in Prague.

## Introduction

The sixth International Symposium on Pheochromocytoma (ISP2022) took place in Prague, Czech Republic, from 19 to 22 October 2022, 5 (and not the usual 3) years after the previous edition held in Sydney, Australia, in 2017, due to delays caused by the COVID-19 pandemic. In the intervening years, two international meetings related to pheochromocytomas (PCC) and paragangliomas (PGL) took place, Multiple Endocrine Neoplasia Workshop 2019 (in Houston) and 2021 (virtual). In 2019, the session dedicated to the metastatic forms of PCC and PGL was reported in a special issue of *Endocrine-Related Cancer* (Dahia *et al.* 2020). Here we summarize some key insights on the genetics of these tumors published or presented over the past years, along with our predictions for future progress in this active field of research.

### New WHO classification

The World Health Organization (WHO)/The International Agency for Research on Cancer (IARC) has historically defined PCC and PGL as distinct entities. The fifth series of the WHO classification of endocrine and neuroendocrine tumors published in 2022 describes clearly PCC as part of the PGL family of tumors by defining PCC as a neuroendocrine neoplasm that originates from chromaffin cells of the adrenal medulla and as an intra-adrenal PGL (Mete *et al.* 2022). A sympathetic PGL is defined as a neuroendocrine neoplasm that develops from neural crest-derived progenitors in paraganglia associated with the prevertebral and paravertebral sympathetic chains, sympathetic nerve plexuses, and sympathetic nerve fibers. Parasympathetic PGLs are well differentiated non-epithelial neoplasms derived from paraganglion cells of the autonomic nervous system. In this review, we will adopt this new nomenclature.

In the absence of clear features that can predict metastatic behavior, all patients with PGL are currently considered to have a lifelong risk of metastases; therefore, conceptually all PGLs of any location are considered potentially metastatic. On the other hand, it is worth noting that the identification of metastases is especially complex in patients with germline predisposition syndromes, as multiple lesions may represent multifocal primary tumors rather than metastatic spread. The review also notes that even the identification of PGL tissue in unusual locations such as the lung or liver does not necessarily imply the diagnosis of metastases, as these may be primary paraganglial locations. The value of sustentacular cells and Ki67 labeling as prognostic features is also discussed in this new classification. A TNM staging system for PGLs (adrenal and extra-adrenal sympathetic PGLs), introduced in the eighth edition of the AJCC Cancer Staging Manual, has now been included in the WHO classification. From a molecular perspective, WHO experts report that germline mutations in *SDHB* confer the highest risk of metastasis and that mutations in *ATRX*, or *SETD2*, high total somatic mutation burden, *MAML3* fusion genes, altered WNT pathway, and *TERT* activation have also been associated with increased metastatic risk (Mete *et al.* 2022).

## The PGL genetic testing in routine practice changes the patients’ outcome

In 2020, the working group on endocrine hypertension of the European Society of Hypertension clearly stated that genetic studies should be considered for any patient diagnosed with PGL because these tumors carry the highest known heritability rate of any human neoplasm and because genetic alterations currently explain almost 80% of all cases. Regarding the latter, approximately 40–50% of the cases are explained by germline mutations in one PGL susceptibility gene identified in patients affected by a hereditary form of the disease, and 40–60% of them by somatic mutations identified in the tumor(s) of patients with the sporadic disease (Lenders *et al.* 2020).

The impact of the knowledge of the genetic status at the time of the first PGL diagnosis was evaluated by Buffet and collaborators in a retrospective multicentric study (Buffet *et al.* 2019). The study compared the management and outcome of 221 patients diagnosed with PGL carrying mutations in *SDHx* or *VHL* who were informed of their positive genetic status either within the first year or more than 7 years after the initial diagnosis of PGL. Fewer patients were lost to follow-up in the group who received their genetic result in the first year after diagnosis compared to those who were tested later (9.6 vs 72%, respectively), and during follow-up, the former group developed smaller new PGLs with lower metastatic spread. In addition, patients of this group who developed metastases had a better 5-year survival rate than patients who did not undergo genetic testing at diagnosis (Buffet *et al.* 2019). That study showed for the first time the positive impact of early knowledge of genetic status, especially for those with *SDHB* variants, as this diagnosis triggers a specialized active surveillance screening program for the management of mutation carriers. This impact on the prognostic value of *SDHB*-related PGL was illustrated in the MAPP-Prono study (169 patients with metastatic PGL) whereby the identification of an *SDHB* mutation lost its significance as a prognostic factor of worse overall survival in these closely monitored individuals, supporting the value of active surveillance in these patients (Hescot *et al.* 2019).

The impact of genetic screening was recognized, among other recommendations, as relevant for the management of metastatic and unresectable PGLs by a multidisciplinary panel convened by The North American Neuroendocrine Tumor Society (Fishbein *et al.* 2021).

### New PPGL susceptibility genes

Over the years, new genes related to the susceptibility to develop PGL have been identified. In this regard, it is worth highlighting the power of massive sequencing platforms, which, together with the genomic characterization of these tumors, have made it possible to recognize the genetic drivers of an additional percentage of patients, whose families will now be able to benefit from appropriate genetic counseling and clinical surveillance.

### DNA methyltransferase 3 alpha (*DNMT3A*) gene

*DNMT3A* encodes one of the two *de novo* DNA methyltransferases, DNMT3A and DNMT3B, and is responsible for establishing DNA methylation patterns during embryonic development and gametogenesis in mammals (Bestor 2000). The implication of this epigenetic modifier in the development of PGL was first demonstrated by Remacha and colleagues (Remacha *et al.* 2018), who described a *de novo* germline mutation in *DNMT3A* in a 22-year-old woman diagnosed with nine head and neck (H&N) PGLs. Functional characterization of the variant detected in this patient, c.896A>T; p.Lys299Ile and detailed analysis of the genomic characteristics of the available PGLs supported an impact of this mutation in DNMT3A function. Overall, these experiments suggested a gain-of-function effect, which was consistent with the hypermethylated profile observed in the tumors with this variant. A second study confirmed the role of *DNMT3A* in the susceptibility to develop H&N PGL (Table 1). The patient presented with multiple clinical features, a finding that suggested a heterogeneous phenotypic spectrum related to *DNMT3A* germline variants (Mellid *et al.* 2020).

**Table 1:** Genetic and clinical characteristics of new genes or new presentations of known genes associated with PGL development.

Gene	Inheritance	Locus	Associated tumors/features
*DNMT3A*	Autosomal dominant	2p23.3	Gain-of-function mutations: H&N PGL
*DLST*	Autosomal dominant	14q24.3	PGL (multiple) >> PCC
*SUCLG2*^a^	ND	3p14.1	PCC >>>> pPGL
*MAML3* fusions	Sporadic	4q31.1	PCC
*RET* fusions	Sporadic	10q11.21	PCC

^a^More evidence are needed before considering it as a susceptibility gene.

### Dihydrolipoamide S-succinyltransferase (*DLST*) gene

*DLST* encodes the E2 subunit of the mitochondrial α-ketoglutarate (αKG) dehydrogenase complex, which catalyzes the overall conversion of αKG to succinyl-CoA and CO_2_ in the tricarboxylic acid (TCA) cycle. Mutations in DLST cause the PGL7 tumor predisposition syndrome (OMIM 618475) and have been found in 0.6–3% of PPGL patients. All *DLST*-related patients described so far were diagnosed with multiple tumors in the thoracoabdominal region, without mutations in other PPG-related genes (Remacha *et al.* 2019, Buffet *et al.* 2021) (Table 1). PGLs harboring these *DLST* mutations display altered methylation and transcriptional profiles similar to those observed in *EPAS1*-mutated tumors, suggesting a connection between DLST functional abrogation and pseudohypoxia.

### Succinate-CoA ligase GDP-forming subunit beta (*SUCLG2*) gene

Succinyl-CoA ligase is a TCA cycle enzyme which catalyzes the reversible conversion of succinyl CoA and adenosine diphosphate (ADP). It is composed of a heterodimer comprising a subunit encoded by *SUCLG1* and an ATP-forming encoded by *SUCLA2* or a GTP-forming subunit encoded by *SUCLG2*. Among 352 patients with PGL, 1 frameshift and 7 missense variants were recently identified in the *SUCLG2* gene (Hadrava Vanova *et al.* 2022), but this first report suffered limitations (no familial aggregation, two *SUCLG2* variants classified as benign or likely-benign variants due to their frequency in gnomAD, incomplete functional studies and lack of evidence for loss of heterozygosity, etc), which were described in the editorial accompanying the paper (Ney & Stewart 2022). Thus, additional studies are still needed before considering *SUCLG2* as a new PGL susceptibility gene (Table 1).

## News from ‘old’ PGL susceptibility genes

### Kinesin family member 1B (*KIF1B*) gene

The *KIF1B* gene has been suspected to be a PGL susceptibility gene in few reports, but its involvement is still debated. Cardot-Bauters and collaborators published in 2008 a family carrying a *KIF1B* missense variant but without a second mutation in the other allele or loss of heterozygosity at the somatic level (Yeh *et al.* 2008). They recently extended their study because one brother of the proband, who did not carry the *KIF1B* variant, developed a bilateral PCC at 31 years. A *MAX* variant was identified in the germline DNA of that patient but also in all his relatives affected by PCC suggesting that the genetic susceptibility to PCC is linked to the MAX variant rather than to the *KIF1B*’s one in this family (Cardot-Bauters *et al.* 2008). While evidence for the role of *KIF1B* in neural crest-related tumorigenesis and in neuroblastomas seems to be supported in independent studies (Fell *et al.* 2017), the Cardot-Bauters *et al*. report further strengthens the view that *KIF1B* is probably not a PGL susceptibility gene.

### Endothelial PAS domain protein 1 (*EPAS1*) gene

Somatic mutations of the *EPAS1* gene, encoding for the hypoxia-inducible factor 2α (HIF2α) transcription factor, are highly prevalent in PGLs. Notably, these mutations are detected in patients with congenital cyanotic heart disease at a higher than 10-fold rate compared with the frequency of *EPAS1* mutations in sporadic PGL (~90% vs ~6–7%), suggesting that these mutations are under selective pressure in the specific clinical/environmental conditions experienced by these patients (Vaidya *et al.* 2018, Ogasawara *et al.* 2022). Of high translational relevance, the first description of a sustained therapeutic response was recently reported in a patient with polycythemia and multiple inoperable PGL caused by a mosaic germline *EPAS1* variant who was treated with belzutifan, a selective small-molecule inhibitor of HIF2α (Kamihara *et al.* 2021). This experience should encourage molecular geneticists to investigate such variants in tumoral DNA and also in the germline DNA (in search for mosaicism) by deep sequencing or digital droplet PCR and should prompt the design of new trials to determine the efficacy of this drug for *EPAS1*-mutated states (Toledo *et al.* 2022).

### Refined methodology to improve genetic variant identification

Over the past few years, technical advances in next-generation sequencing methods applied to germline and tumoral DNA associated with the decreased cost of sequencing have allowed the introduction of whole exome or genome sequencing in research and also in routine practice. Non-classical pathogenic variants and rare genetic variants became more easily accessible and were reported in patients for whom the causative germline or tumoral variant was still unknown (Ben Aim *et al.* 2022). For instance, the diagnosis of germline mosaicism in PGL susceptibility genes became possible in the same assay, by identification of a variant in a minority of reads in germline DNA and in the majority of reads in its matched tumoral DNA. Whereas a minor peak on electropherogram of Sanger sequencing can be misinterpreted as an artifact, droplet digital PCR allows to precisely quantify a low level of mosaicism in DNA extracted from leukocytes or other tissues. In this way, the first case of constitutional mosaicism of *SDHB* mutation was reported in a young patient with a norepinephrine-producing extra-adrenal PGL (Cardot-Bauters *et al.* 2019).

Intronic deep mutations were reported in PGL susceptibility genes. An international effort from the US and Europe brought new evidence for the pathogenicity of deep intronic variants in the *VHL* gene in a cohort reassembling 1167 patients with previous negative genetic testing. Six different genetic variants were discovered in a cryptic exon of *VHL*, named E1’, which was previously identified in deep intronic sequence but not usually included in the target gene panels (Buffet *et al.* 2020). A study from Australia demonstrated the activation of an exonic splicing enhancer by a genetic variant located in the +74 position of the *SDHC* gene (De Sousa *et al.* 2020). Other deep intronic pathogenic variants would likely be more detected and involved in PGL pathogenesis in the upcoming years. Synonymous variants at non-canonical splice sites but which may nonetheless impair splice, such as in the case of the *VHL* gene, were demonstrated as being pathogenic in patients affected by a von Hippel Lindau disease or familial erythrocytosis (Lenglet *et al.* 2018, Flores *et al.* 2019, Liu *et al.* 2020, Buffet *et al.* 2020).

Incidental genetic findings were also discovered due to these highly sensitive technologies. This was the case in two recently published case reports: the first described the simultaneous identification of germline mutations both in *SDHB* and in *TP53* in a patient with metastatic PCC (Gniado *et al.* 2020), and the second reported germline mutations in the *FLCN* and *SDHB* genes in a patient with metastatic renal cell carcinoma (Boland *et al.* 2020). Noteworthy, these discoveries raise new challenges for the practice of genetic counseling and recommendations on the surveillance of patients and relatives.

### Fusion genes

Recombinant fusions have only recently begun to be recognized and evaluated in PGL. Rare fusion genes, involving *MAML3*, *BRAF*, *NGFR*, and *NF1*, were first discovered by RNA sequencing in The Cancer Genome Atlas PPGL study (Fishbein *et al.* 2017). Fusions recurrently involving the *MAML3* transcription factor, especially the *UBTF*::*MAML3* fusions, were associated with a novel molecular cluster that had not been previously recognized in PGL oncogenesis. The *UBTF*::*MAML3* fusion leads to the expression of Wnt targets and appears to be associated with an aggressive phenotype (Alzofon *et al.* 2021). *UBTF*::*MAML3,* but also *EWSR1*::*CREM* fusion gene, which was recently reported in a patient with a metastatic PGL should be further investigated as prognostic biomarkers (Javaid *et al.* 2023).

The *RET* gene is a well-established PGL susceptibility gene as part of Multiple Endocrine Neoplasia type 2A and type 2B syndromes, and less commonly as a somatically mutated oncogene, but only recently was it detected as part of recombinant fusion in PGLs (Mweempwa *et al.* 2021, Estrada-Zuniga *et al.* 2022). These fusions are distinct from conventional *RET* rearrangements detected in epithelial cancers such as lung and thyroid (Grieco *et al.* 1990, Kohno *et al.* 2012, Santoro *et al.* 2020) in the positioning of the fusion partners. In PGLs, *RET* is the 5′ partner of the fusion, while in epithelial tumors, it is invariably positioned as the downstream partner of the fusion. Despite this distinct recombination architecture, *RET* fusions in PGLs share other features of the more typical *RET* recombinant proteins: they lead to constitutive activation of *RET* and its downstream effectors, endow target cells with oncogenic phenotypes (Ou & Zhu 2020, Santoro *et al.* 2020, Estrada-Zuniga *et al.* 2022), and are responsive to highly selective, clinical grade RET inhibitors selpercatinib and pralsetinib (Subbiah *et al.* 2018, Wirth *et al.* 2020, Mweempwa *et al.* 2021, Thein *et al.* 2021, Estrada-Zuniga *et al.* 2022). These findings suggest that the identification of gene fusion involving the *RET* gene should open the way to treatment with RET inhibitors in patients with metastatic or inoperable PGL. Due to its prognostic and therapeutic value, the search for fusion genes in PGL at the tumor level by transcriptome or whole genome sequencing should be added to the genetic testing arsenal of PGLs that remain without a recognizable driver event, especially those belonging to the kinase cluster.

### New tools for accurate variant’s classification

Nowadays, the major challenge for the molecular geneticists in charge of PGL diagnosis remains to correctly classify molecular variants identified by NGS. In 2017, the NGS in PPGL (NGSnPPGL) Study Group (ENS@T/PRESSOR) published standardized recommendations and initiated an international effort to collect, annotate, and classify variants in order to develop gene-centric curated database of PGLs (The NGS in PPGL (NGSnPPGL) Study Group *et al.* 2017). Following this initiative, 223 *SDHB* variants from 737 patients were collected worldwide and manually curated by a panel of experts from the NGSnPPGL Study Group who established a consensus classification. This curation reduced by half the variants initially classified as variants of unknown significance (Ben Aim *et al.* 2022). The *SDHB* variants classified by these experts are now freely available and publicly accessible via the Leiden Open Variation Database (LOVD) system (https://databases.lovd.nl/shared/genes/SDHB).

There have also been advances in our annotation of *TMEM127* variants. Recent structure-functional studies revealed additional features of *TMEM127*, including a fourth transmembrane domain and an endocytic domain (Flores *et al.* 2020). These findings provided the basis for preliminary evaluation of a new classification of variant pathogenicity in 111 carriers and support the location of nonconserved missense mutations in transmembrane domains as a likely feature of pathogenic variants (Armaiz-Pena *et al.* 2021) Most of these variants have been deposited in LOVD (https://databases.lovd.nl/shared/genes/TMEM127).

### New insights for asymptomatic mutation carriers

An international panel of experts has established a consensus statement in following the Delphi method focused on the management of *SDHx* asymptomatic mutation carriers detected by familial genetic testing. An algorithm for screening and follow-up was proposed during adulthood and childhood. The experts proposed first screening at an earlier age (6–10 years old) for asymptomatic *SDHB* mutation carriers than for carriers of mutations in the other SDHx genes (10–15 years old) and recommended using magnetic resonance imaging as first-line imaging in children. If a *SDHx* mutation carrier never developed any tumor related to SDH deficiency, screening tests could be delayed to every 5 years after 70 years of age and follow-up could be stopped at 80 years of age (Amar *et al.* 2021). A retrospective multicentric study reported 249 asymptomatic SDHx mutation carriers who benefited from at least 1 imaging work-up. Imaging screening detected tumors in 20% of asymptomatic SDHx mutation carriers with a median age of 41 years old (11–86) (Saie *et al.* 2021). Similar proportions have been reported in other two independent multicenter studies (Greenberg *et al.* 2020, Davidoff *et al.* 2022), demonstrating that current SDHx screening protocols are effective at identifying SDHx-related tumors. The benefits of surveillance of asymptomatic mutation carriers starting in childhood have been reported by Vibert and collaborators in the context of the von Hippel-Lindau disease, for which genetic testing is recommended starting at 5 years of age. In a small series of 16 children diagnosed as *VHL* mutation carriers, follow-up examinations performed in a specialized expert network detected 11 tumors in 6 children but all had a favorable outcome (Vibert *et al.* 2022).

### ISP2022 highlights in approaches and models for PGL research

A recent multi-institutional research effort led by Richard Tothill examined a group of 30 PGLs carrying mutations in various susceptibility genes at single nuclei transcriptome resolution (Zethoven *et al.* 2022). Different expression subgroups were identified corresponding to former recognized transcriptomic clusters driven by genotype: C1A (*SDHx*), C1B1 (*VHL*), C1B2 (*EPAS1*), C2A (Kinase), C2B1 (*MAX*), and C2B2 (*MAML3*). In addition, *VHL*, *SDH*, and *EPAS1*-mutated groups were enriched in stromal cells and tumor nuclei and showed enhanced hypoxia-related signaling. Intriguingly, tumors with *MAML3* fusions, often linked to aggressive outcomes, showed a high expression of *VEGFA* and *EPAS1* suggesting a HIF-pathway activation by still unknown mechanisms. Metastatic *SDHx*-related PGLs displayed an increase in proliferation markers and a reduced number of Schwann-cell-like cells. The orphan receptor *GPR139* emerged as one of the overexpressed genes in metastatic tumors and should be further investigated as a potential target for treatment.

The scarcity of experimental models in PGLs has been an important unmet need in research. New and promising research models were presented at ISP2022, including two new *Sdhb*-deficient mouse strains (Armstrong *et al.* 2022, Gupta *et al.* 2022). Another emerging model of patient tumor-derived organoid cultures that may be amenable to drug screens may provide interesting information on the therapeutic profile of PGLs (Dahia, Soragni *et al*., unpublished observations). Follow-up work in these models is highly anticipated.

## Future of clinical trials in PGL

Lastly, the next big frontier in PGL research will come from new therapies based on strong biological rationale and which are being tested in new clinical trials. The results of the first international randomized study in metastatic progressive PGL (FIRSTMAPPP, NCT01371201) investigating sunitinib (37.5 mg/day) or placebo were first presented during European Society of Medical Oncology Congress 2021 and at ISP2022. The median PFS was 8.9 months in the sunitinib arm vs 3.6 months in the placebo. This academic double-blind trial, in which 78 patients were enrolled, provided the highest level of evidence available thus far and established sunitinib as the first-line option for affected patients with progressive metastatic PGL (Baudin *et al.* 2021). On the heels of its approval by the FDA for the treatment of *VHL*-related tumors (Jonasch *et al.* 2021), an international, multi-institutional phase 2 clinical trial of belzutifan was launched for patients with locally advanced or metastatic PPGLs (NCT04924075). This trial recently completed its recruitment, and the PGL field expects with great anticipation the results of this and other trials grounded by preclinical research to guide the future of PGL treatment.

## Declaration of interest

The authors declare that there is no conflict of interest that could be perceived as prejudicing the impartiality of this review.

## Funding

A-P G-R receives funding support from the European Commission FP7 Research and Innovation Funding Program for 2007–2013 (n° 259735), the European Union's Horizon 2020 research and innovation program under grant agreement No 633983, the Institut National du Cancer and Direction Générale de l’Offre de Soins (DGOS), Programme de Recherche Translationnelle en cancérologie (PRT-K 2014, COMETE-TACTIC, INCa_DGOS_8663) and the Cancer Research for Personalized Medicine – CARPEM project (Site de Recherche Intégré sur le Cancer- SIRIC). Her team is supported by the Ligue Nationale contre le Cancer (Equipe Labellisée). M R is funded by grants from the Instituto de Salud Carlos III (ISCIII), through the ‘Acción Estratégica en Salud’ (AES) (project PI20/01169), Paradifference Foundation, and the Pheipas Association. P L M D is supported by Hayes Distinguished Endowed Chair in Oncology, NIH-NIGMS (GM114102), NIH-NCI (CA264248), Neuroendocrine Tumor Research Foundation, VHL Alliance, and the University of Texas System STARS award.
